# Bilateral Phyllodes in a 12-Year-Old Adolescent: Report of a Rare Case

**DOI:** 10.7759/cureus.27567

**Published:** 2022-08-01

**Authors:** Sneha H Kenjale, Sajika P Dighe, Raju K Shinde, Sangita D Jogdand, Dewang B Ghode

**Affiliations:** 1 Surgery, Jawaharlal Nehru Medical College, Datta Meghe Institute of Medical Sciences, Wardha, IND; 2 General Surgery, Jawaharlal Nehru Medical College, Datta Meghe Institute of Medical Sciences, Wardha, IND; 3 Pharmacology, Jawaharlal Nehru Medical College, Datta Meghe Institute of Medical Sciences, Wardha, IND

**Keywords:** breast reconstruction, mastectomy, premenarchal female, bilateral, adolescent, giant fibroadenomas, breast neoplasms, phyllodes tumor

## Abstract

Phyllodes tumors, previously known as cystosarcoma phyllodes, are fibroepithelial tumors that consist of epithelial and cellular stromal components. These tumors have a predilection to recur after wide local excision and to attain massive sizes. They account for less than 0.5% of cases of all breast neoplasms and are primarily found in the third to fourth decade of life, and rarely in adolescence. However, less than 25 cases are reported to date wherein this tumor is found in adolescent females, with this case being one of those. This is a report of a rare case of a 12-year-old premenarchal female with bilateral phyllodes tumor, highlighting its peculiarities, diagnostic features, and surgical management in view of the patient’s age and quality of life.

## Introduction

Breast is a modified sweat gland that lies in the pectoral region. Out of the several congenital, inflammatory, and neoplastic lesions, phyllodes tumor or cystosarcoma phyllodes accounts for 0.3-0.5% of tumors [1], with the majority of them being benign. In 1838, Johannes Muller described it as a tumor with a fleshy, greyish white, leaf-like mass, hence the name phyllodes (phyllos - leaf, Greek) [2]. However, in 1982, WHO declared “phyllodes” as the most appropriate term out of the other 60 synonyms of the lesion [3]. It is commonly noted in the third to fourth decades of life at an incidence of 2.1 per million [2,3], out of which, cases of adolescent, premenarchal females in the bilateral breast are negligible, i.e. less than 0.1%.

Clinically, it presents as a palpable, rapidly enlarging mass ranging from 4 cm to 20 cm [4,5]. The skin over the lesion is stretched and shiny with a bosselated feel and the chest wall occasionally shows dilated engorged vessels. Mass is not fixed to the skin or the underlying structures and lymphatic spread is rare. Depending on the mitotic activity, cellular atypia, and infiltration at tumor borders, 70% of the phyllodes are benign, 20% are malignant, and the rest 10% remain borderline [4,6]. Malignant ones are aggressive in nature and have the tendency to recur with survival rates of 91%, 89%, and 89% at 5, 10, and 15 years, respectively [3]. Initial investigation in a young female includes an ultrasound (USG) followed by fine-needle aspiration cytology (FNAC) and image-guided biopsy. MRI is useful in large masses to evaluate the extent, margin, and status of the surrounding tissue [7].

Definitive management in such cases is surgical, either in the form of simple mastectomy or wide local excision along with at least 1 cm margin [3] and aiming for breast reconstruction wherever possible. Adjuvant radiotherapy and hormonal modulation can be tried in a few cases. This case particularly finds rarity due to the massive size and bilateral representation in a premenarchal, unmarried, adolescent female wherein definitive surgical treatment is a difficult decision. 

## Case presentation

A 12-year-old female came with chief complaints of bilateral enlargement of the breast since one year and pain since 15 days. As narrated by the patient herself and her mother, she was apparently alright one year back when she noticed a lump in both her breasts while bathing. The lump in the right breast started in the upper outer quadrant and the left breast lump was first felt in the retro-areolar region. Its size rapidly progressed, such that it was approximately of size 2×3 cm at the start, which progressed to the current size of 10×10 cm. The lump was painless initially but became painful with associated heaviness in the chest region since eight months. The pain was localized in both the breasts and was non-radiating. There was no history of fever, discharge from the nipple, retraction of the nipple, ulceration over the breast, or significant weight loss. She did not take any treatment previously and her history is not significant. There is no history of breast malignancy in the family. Her sleep, bowel bladder habits, and appetite are normal. However, the pain became worse in 15 days to an extent that it hampered her daily activities like bathing and changing clothes, following which she approached our hospital.

**Figure 1:**
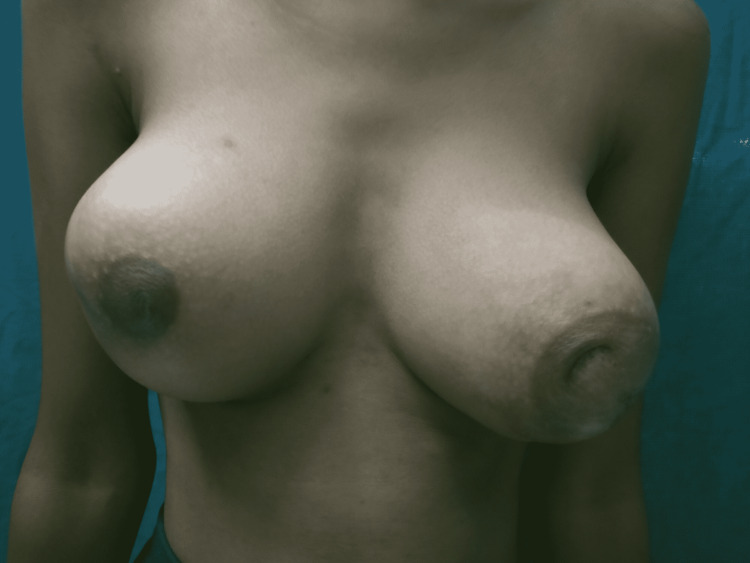
Clinical photograph of the examination showing asymmetrical enlargement of the bilateral breast and distortion of the nipple-areola complex on the left side.

**Figure 2:**
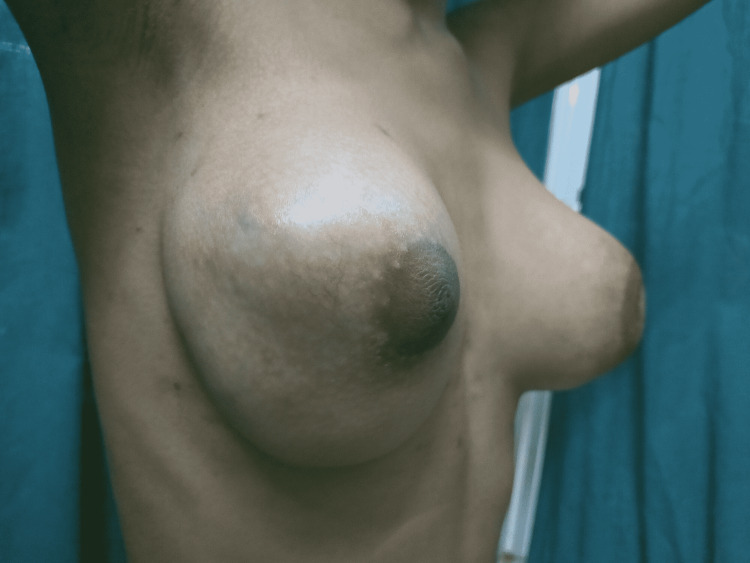
Clinical photograph of the examination showing bilateral breast enlargement without ulceration, fungation, or discharge from the nipple and the intact nipple-areola complex on the right side.

Her general and systemic examination was normal. Local examination revealed bilateral enlargement of the breast with a lump of size 8×10 cm approximately. There was no puckering, dimpling, ulceration, or fungation of the breast. The skin over the breast appeared to be tense without any engorged veins. The left nipple was at a lower level than the right one and the nipple-areola complex on the left side was completely distorted and destructed. There was no discharge from the nipple, and the arms, thorax, axilla, and supraclavicular fossa appeared to be normal. Local temperature was raised with marked tenderness bilaterally. It was bosselated and was firm in consistency. The lump was not fixed to the chest wall and no lymph nodes were palpable. A clinical photograph of the local examination is shown in Figures 1, 2.

**Figure 3:**
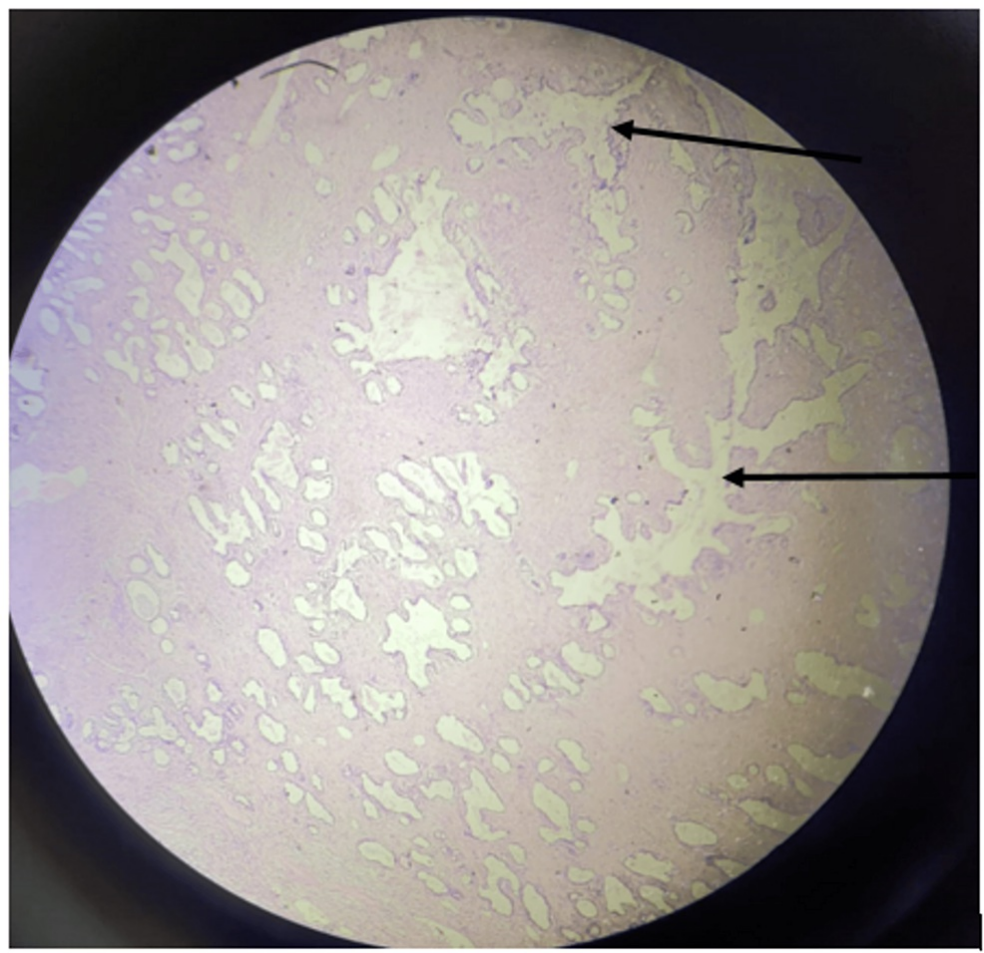
Microscopic photograph of histopathology of excised phyllodes tumor. Arrow heads showing "leaf"-like pattern suggest phyllodes tumor (hematoxylin-eosin, scanner).

**Figure 4:**
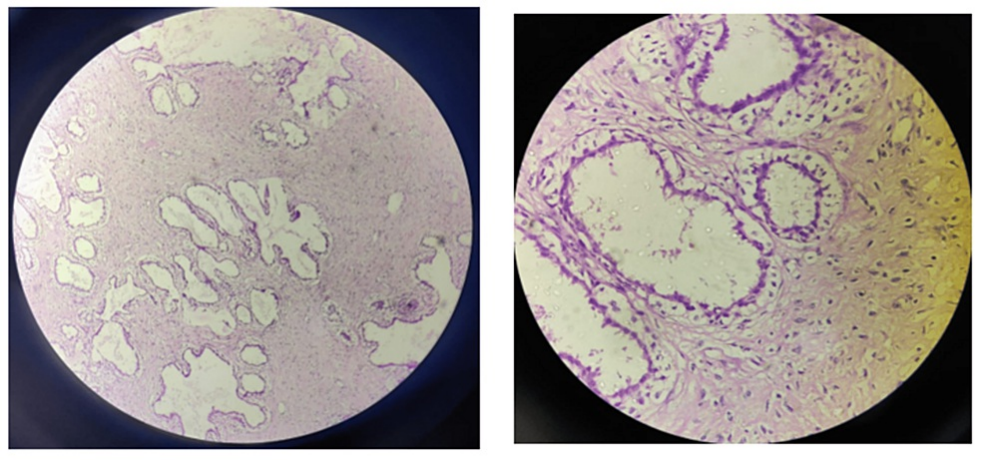
Minimal stromal atypia and cellularity, suggesting benign phyllodes tumor (hemotoxylin-eosin, 10x and 40x).

The USG revealed a large, heterogeneous, hyperechoic lesion of size 10×6 cm on the right side and 8×7 cm on the left side, without any spiculations or calcifications. The lesion involved all four quadrants and was suspected to be either a giant juvenile fibroadenoma or a benign phyllodes tumor. FNAC was conducted from both sides, which was suggestive of a benign phyllodes tumor. A definitive diagnosis was obtained by biopsy and histopathology confirmed benign phyllodes tumor on both sides, as seen in Figures 3, 4.

**Figure 5:**
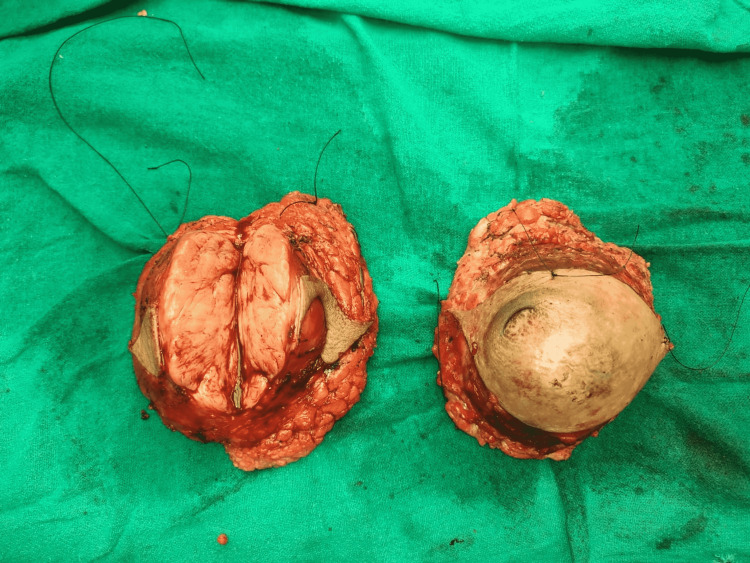
Intraoperative photograph of the excised bilateral phyllodes tumor.

**Figure 6:**
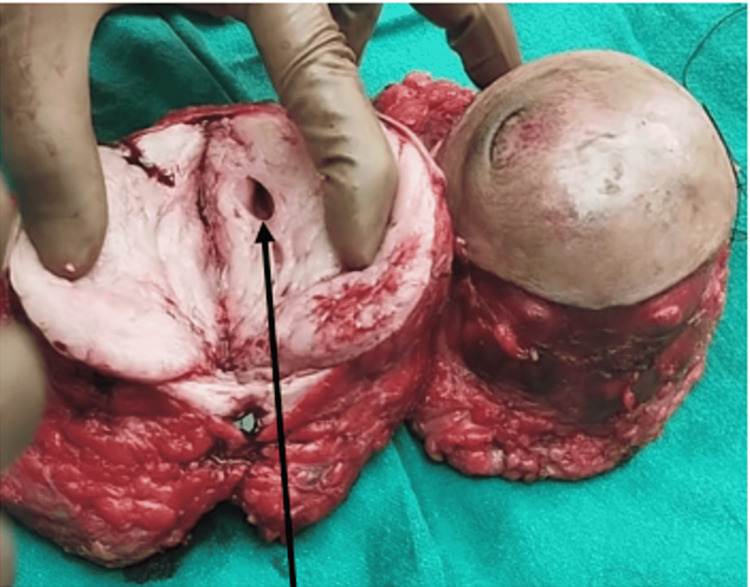
Intraoperative photograph of the excised phyllodes tumor where the arrow head is showing cystic spaces inside the tumor.

**Figure 7:**
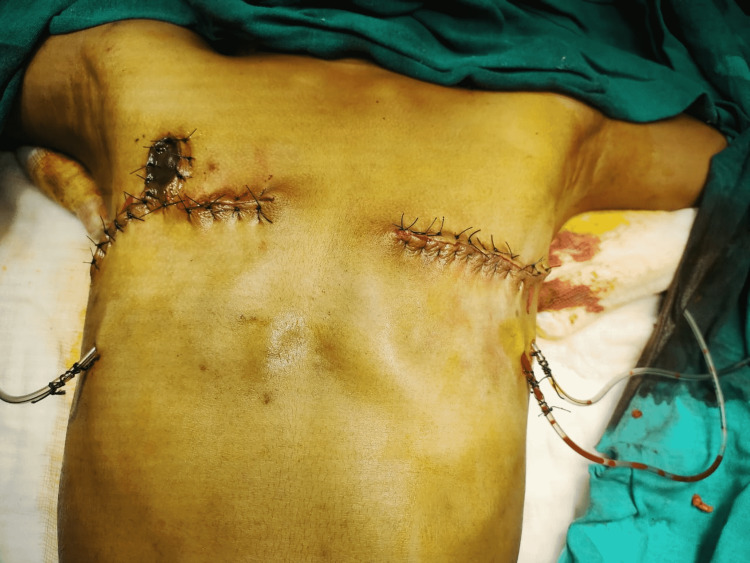
Post-mastectomy photograph showing preserved nipple-areola complex on the right side.

Ideally phyllodes tumor is dealt with by wide local excision but in our case the lumps in the bilateral breast were extremely huge, making it impossible to conduct a breast-sparing surgery. Also, chances of recurrence in the later phase would have been increased in case of breast-sparing surgery. Hence, bilateral mastectomies were performed. An intraoperative photograph of the tumor is seen in Figures 5, 6. As the nipple on the left side was distorted, it was removed but the nipple-areolar complex on the right side was spared as shown in Figure 7. The patient was counseled emotionally as the loss of breasts on both sides was mentally devastating. Reassurance regarding breast reconstruction surgery was given to the patient and her parents.

## Discussion

Phyllodes tumor, although accounting for less than 1% of breast neoplasms, should be always kept in mind as a differential diagnosis whenever a patient comes with a rapidly growing mass in the breast. Its median age is around 45-50 years but still is a rare possibility in adolescents as well [1,7]. Until 2016, only 20 such cases of adolescent phyllodes have been reported [1]. Ninety-five percent of pediatric breast masses are benign and can be conservatively treated. However, 0.1% of these can convert into malignant phyllodes [1,2].

Clinical suspicion, case-based individualized approach, and multimodal investigations lead us to diagnosis, as seen in a 19-year-old girl, in the case report by Prihantono et al., complaining of rapid enlargement of a unilateral breast lump to size 20×16 cm just in three months' span [2]. Similarly, in Tanzania, an adolescent with a huge, painful, ulcerative breast mass was diagnosed with a phyllodes tumor of size 31 cm [5]. In some cases, only pain is noted as a chief complaint, as seen in a 17-year-old girl whose breast size was relatively the same as compared to one another [4]. In one of the case reports by Vargas C et al., a 12-year-old had serous nipple discharge along with phyllodes, and another similar case reported by him developed skin ulceration and pressure necrosis as it was left untreated for three years due to family negligence [6,7].

The presentation in phyllodes tumors can be varied; hence, clinical, radiological, FNAC, or tissue biopsy evaluation followed by subsequent excision is a must for definitive management. As developing pediatric breast tissue is highly sensitive to ionizing radiation, ultrasonography is better than mammography in this age group [8]. CT scan is used to look for thoracic abnormalities whereas MRI can be used to check the vascular and lymphatic status prior to surgery [8].

On USG, it is difficult to differentiate between fibroadenoma and phyllodes as there are several overlapping features; however, phyllodes show more cystic areas than fibroadenoma on an USG [1,6,8]. One such case reported that juvenile fibroadenoma converted into a borderline phyllodes tumor in an 18-year-old [9]. A case of phyllodes in an 11-year-old girl from Mumbai and another in a 12-year-old from Chile were investigated to reveal that both of their USG and FNAC reports were suggestive of giant fibroadenoma [1,6]. Similar findings were reported in our case.

It is essential to delineate the differences between phyllodes and fibroadenoma as their plan of management is different. To categorize the lesion as juvenile fibroadenoma, Stanford has issued guidelines as follows: circumscribed and rarely multiple, biphasic stromal and epithelial process lacking a leaf-like growth pattern in a uniformly hypercellular stroma, a lack of atypical features, and a stroma-like periductal increase in cellularity, stromal overgrowth, and cytologic atypia, as well as a mitotic rate <3/high-power field, frequent epithelial and myoepithelial hyperplasia, and an age of between 10 and 20 years [1,10], whereas Stanford defines giant fibroadenoma as tumors more than 500 g [1,10]. Some of the differences between fibroadenoma and phyllodes are tabulated in Table 1 [1].

**Table 1:** Differences between fibroadenoma and phyllodes tumor.

Differentiating characteristics	Fibroadenoma	Phyllodes tumor
Size and growth	Slow growing	Rapid growth
Histopathology	Fibrous stroma consisting duct-like spaces	“Leaf pattern”
Mitotic figures, stromal growth, and infiltration	Not present	Present
Treatment (surgical)	Enucleation	Wide local excision/mastectomy
Local recurrence	Uncommon	Very common
Metastasis	Not known	Seen in malignant phyllodes

Core needle biopsy is the final investigation to get the definitive diagnosis [8]. Depending on mitotic figures, cellular atypia, and infiltration of tumor margin, biopsy results are categorized into benign (70%), malignant (20%), and borderline (10%) by WHO, as in Table 2.

**Table 2:** Classification of phyllodes tumor on the basis of biopsy reports.

Classification according to biopsy reports	Margin	Mitoses per 10 high-power field	Stromal overgrowth	Stromal atypia
Benign phyllodes	Pushing	<5	Not seen	Minimal
Borderline phyllodes	Pushing or infiltrating	5-10	Present	Moderate
Malignant phyllodes	Infiltrating	≥10	Present	Severe

Depending on the histopathology reports, as shown above, complications are foreseen and the plan of management is outlined. Benign and borderline variants pose a lesser risk than the malignant ones. If left untreated, they undergo pressure necrosis and ulceration [3] or can lead to secondary infection. In rare cases, these tumors can metastasize to lungs and long bones. Only one such reported case from Madhya Pradesh, India, was of an 18-year-old girl whose malignant phyllodes tumor was associated with isolated brain metastasis [11].

The definitive line of management of phyllodes, be it benign, borderline, or malignant, remains surgical wide local excision with a 1 cm margin or simple mastectomy, as the lymphatic spread is uncommon to axillary lymph nodes. However, a study of 19 reported phyllodes cases by Leveque et al. suggests that wide local excision should be performed more often and simple mastectomy should be reserved for recurrent cases [12]. Radiotherapy and hormonal modulation give temporary regression in size, whereas chemotherapy has no proven role.

In cases of large lumps of benign phyllodes, mastectomy is indicated, as seen in our case. Phyllodes tumors tend to recur and studies have reported that such patients have an increased risk of breast carcinoma in the future if not managed appropriately; hence, simple mastectomy forms a preferred choice here. The patient in our case underwent simple mastectomy bilaterally, wherein the nipple-areola complex on the right side was preserved. She will be planned for breast reconstruction surgery as soon as she attains her menarche. Prosthetic reconstruction also can be done immediately, provided the patient has already achieved her menarche [12]. In the case report by Serhan et al., an 11-year-old girl who had already attained her menarche was treated by a total skin-sparing mastectomy via an elliptical peri-areolar incision and immediate breast reconstruction with adjustable saline implants [8,13], whereas breast reconstruction was successfully done in cases even where the bottom skin of the nipple was involved causing nipple retraction. The surgery involved mobilization of the areola by skin flaps followed by insertion of implants [14].

## Conclusions

With less than 25 cases reported to date, breast neoplasms in the adolescent age group are extremely rare. Our case report is of a 12-year-old premenarchal girl, who came with a bilateral phyllodes tumor. Such a unique and uncommon presentation requires vigilant diagnosis and prompt treatment. Definitive management in these cases is surgical, hence the patient underwent a simple mastectomy and will undergo breast reconstruction once she attains her menarche, for a better quality of life. However, the massive size of the tumor and the bilateral representation in a premenarchal, unmarried, adolescent female made the decision of surgical treatment even more challenging. Case-based diagnostic approach and reconstructive measures taken in this case may provide useful insight for the clinicians and help them formulate more appropriate management.
